# Navigating the Landscape of Direct-to-Consumer Telehealth Services

**DOI:** 10.7759/cureus.79173

**Published:** 2025-02-17

**Authors:** Deesha D Desai, Ya-Han (Crystal) Zhang, Ambika Nohria, Vartan Pahalyants, Ata S Moshiri, Kristen I Lo Sicco

**Affiliations:** 1 Department of Dermatology, University of Pittsburgh School of Medicine, Pittsburgh, USA; 2 The Ronald O. Perelman Department of Dermatology, NYU Grossman School of Medicine, New York, USA

**Keywords:** alopecia, dermatology, direct-to-consumer, dtc, telehealth

## Abstract

Direct-to-consumer (DTC) telehealth platforms have enhanced healthcare accessibility and convenience, particularly for Individuals in remote areas or those with mobility limitations. These platforms offer virtual medical consultations and prescription services, improving access to treatment for conditions such as hair loss, anxiety, depression, and sexual dysfunction. However, concerns regarding limited physician oversight, lack of transparency in provider qualifications, privacy risks, and potential financial and legal vulnerabilities highlight the need for stricter regulations. Addressing these issues is crucial to ensure patient protection and trust as the use of DTC telehealth services continues to grow.

## Editorial

With the introduction of telehealth services, direct-to-consumer (DTC) companies have emerged as prominent healthcare platforms. These platforms offer accessibility and convenience for conditions, including hair loss, anxiety, depression, and sexual dysfunction. However, while these platforms expand healthcare access, they require closer regulation to ensure patient protection.

Benefits of DTC platforms

DTC platforms enhance healthcare accessibility and convenience by virtually connecting patients with healthcare services, including prescription writing, potentially eliminating the need for traditional in-person visits. This is particularly beneficial for individuals in remote areas or those with mobility limitations, providing access to necessary medications and resources that might otherwise be difficult to obtain [1]. Furthermore, these platforms may save time and money for select patients [1].

Identifying the issue

A central concern with DTC services is liability within terms and conditions agreements. Frequently, patients consent to services without a thorough review, leaving them vulnerable to medical oversight as well as financial and legal risks [2]. These vulnerabilities emphasize the necessity for greater transparency.

Medical concerns

Medically, a key issue lies in the lack of robust physician oversight [2]. Patients often navigate side effects without the guidance of an involved healthcare professional. This unilateral responsibility for medical decision-making places a heavy burden on patients, who may lack the necessary healthcare literacy to fully comprehend treatment implications.

Additionally, the lack of transparency in provider qualifications raises concern. Patients are often unsure about the training of their healthcare provider, raising doubts regarding the care received. This lack of clarity is amplified by the differences in state laws regarding the independent practice guidelines for healthcare providers, raising questions about the medical providers’ abilities to prescribe medications in the region where the patient resides.

Moreover, privacy concerns, particularly the absence of Health Insurance Portability and Accountability Act (HIPAA) coverage, expose patients to the risk of unauthorized disclosure of their private health information. The use of specific language, such as *business associates*, further obscures the nature of the relationship between patients and providers, exacerbating privacy anxieties.

Financial and legal ramifications

Financial complexities emerge as DTC companies may provide medication coverage without frequent consultations, risking unchecked usage [2]. Legal strategies, like clauses preventing class-action lawsuits and capping patient compensation, weaken protections. Furthermore, legal language blurs the line between business and clinical practices, with terms like *individuals* instead of *patients*, undermining patient-physician trust.

Additionally, there is a concerning push for expanded capacity to prescribe and treat. Lobbying initiatives, such as HB546, advocate for prescribing Schedule VI controlled substances via telemedicine [3]. These efforts raise concern for broadening potential avenues medications can be prescribed, possibly bypassing traditional safeguards and thorough clinical oversight.

The reach of companies like hims & hers, as indicated by $872 million in revenue in 2023, underscores the urgency in addressing these issues [4]. Similarly, Ro's revenue reached $300 million in 2023, indicating rapid DTC platform expansion [5]. As these companies expand, it is essential to establish transparency and patient protection guidelines.

Conclusions

In summary, DTC telehealth platforms enhance healthcare accessibility and convenience, especially for individuals in remote areas or those with mobility limitations. However, these benefits come with notable risks, including concerns regarding physician oversight, transparency in provider qualifications, and patient privacy. Financial and legal vulnerabilities also emerge from complex terms and conditions, potentially compromising patient protection and trust.

Given the rapid expansion of DTC platforms, it is crucial to establish robust regulations that ensure transparency and safeguard patient interests. Balancing accessibility with comprehensive oversight and clear communication is essential to maximize the benefits of DTC telehealth while minimizing associated risks.
